# Experimental and Numerical Investigation of Flow Field and Soot Particle Size Distribution of Methane-Containing Gas Mixtures in a Swirling Burner

**DOI:** 10.1021/acsomega.1c04895

**Published:** 2021-12-22

**Authors:** Zari Musavi, Yao Zhang, Etienne Robert, Klas Engvall

**Affiliations:** † Dept. of Chemical Engineering, KTH Royal Institute of Technology, Stockholm SE-10044, Sweden; ‡ Dept. of Mechanical Engineering, Polytechnique Montréal, Montréal QC H3T 1J4, Canada

## Abstract

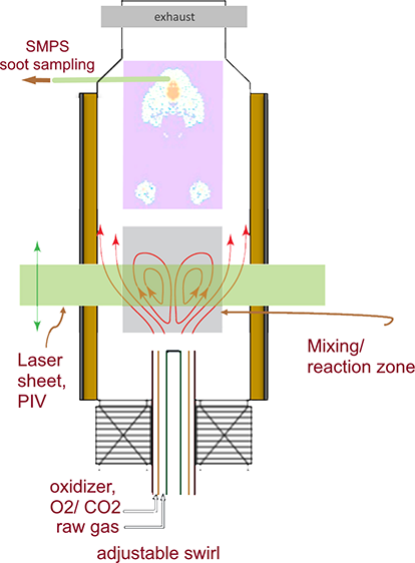

The formation of soot in a swirling flow is investigated experimentally and numerically in the context of biogas combustion using a CO_2_-diluted methane/oxygen flame. Visualization of the swirling flow field and characterization of the burner geometry is obtained through PIV measurements. The soot particle size distributions under different fuel concentrations and swirling conditions are measured, revealing an overall reduction of soot concentration and smaller particle sizes with increasing swirling intensities and leaner flames. An axisymmetric two-dimensional CFD model, including a detailed combustion reaction mechanism and soot formation submodel, was implemented using a commercial computational fluid dynamics (CFD) code (Ansys Fluent). The results are compared with the experiments, with similar trends observed for the soot size distribution under fuel-lean conditions. However, the model is not accurate enough to capture soot formation in fuel-rich combustion cases. In general, soot particle sizes from the model are much smaller than those observed in the experiments, with possible reasons being the inappropriate modeling in Fluent of governing mechanisms for soot agglomeration, growth, and oxidation for CH_4_-CO_2_ mixtures.

## 1 Introduction

Soot formation has traditionally been a problem in diesel internal combustion engines, open fires, and large boilers.^1,2^ Soot particles are produced as a result of incomplete combustion processes in gas phase reactions, where hydrocarbon molecules may start growing, eventually condensing and agglomerating to form dense particles.^3^ Soot formation is generally undesirable; it represents an energy loss from unreacted carbon, although its presence can be beneficial to enhance radiative heat transfer. Soot causes significant harm because of its polluting effects. It is responsible for certain health problems associated with inhaling fine soot particles, as well as changing the planet albedo by deposition on frozen surfaces. Soot formation also brings problems in industrial processes downstream of the combustion itself, such as polymerization and clogging of equipment, which can result in process shutdowns.

Thermochemical or biological biomass conversion processes yield gas mixtures of varying compositions such as syngas and biogas. These gas mixtures can be used as they are but are often processed into hydrocarbons suitable for replacing fossil fuels, such as biomethane and liquid biofuels. Chemical reactions such as partial oxidation (POX)^4,5^ and catalytic reforming play a critical role in such conversion processes. They are, however, very sensitive to soot formation and fouling.

Combustion and soot formation modeling are challenging tasks to begin with, especially when dealing with a complex mixture of combustible gases. In general, a combustion reaction mechanism is developed for specific fuels. Examples include the GRI-3 mechanism,^6^ the Konnov 0.5 mechanism,^7^ and the Petersen mechanism^8^ for methane and natural gas oxidation reactions, the USC II mechanism^9^ for C1–C4, benzene, and toluene oxidation, as well as the LLNL mechanism^10^ for oxidation of C7–C20 hydrocarbons. However, model complexity increases dramatically when the complex molecules involved in soot formation are considered. For instance, a model developed by MIT (296 species and 1322 reactions) includes the formation and destruction of soot particles and PAHs up to three condensed rings in addition to combustion of hydrocarbons up to benzene.^11^ Numerical models implementing these mechanisms in CFD simulations of realistic conditions of burners and POX reactors are very computationally expensive, with experimental datasets essential to assess the validity of the choices made in model development.

Numerous works combining chemical kinetics modeling and experiments in combustion and soot formation in laminar premixed and non-premixed flames have been performed.^12−19^ Model results compared with experiments show that even in simple flames, there are difficulties in predicting soot.^20,21^ These kinetic models are developed and evaluated using programs such as FlameMaster, RMG, REACTION, and ANSYS Chemkin. Although many studies and fundamental research efforts aim to increase the understanding of soot formation mechanisms, combining detailed combustion mechanisms with realistic flow field to capture the effects of choices made in the numerical model on soot inception, growth, and oxidation has seldom been done. Verification of the models is mainly done comparing ignition delay and flame speed in zero- or one-dimensional models and the experiments.^20,22−26^ Gas mixtures encountered in syngas and biogas conversion processes are rarely considered in soot studies, and research efforts are needed to assess the capabilities of available numerical tools.^5,27^

In the present study, experimental and numerical modeling tools were combined to investigate the interactions between soot formation, chemical kinetics, and turbulence in a swirling non-premixed burner geometry. A burner with adjustable swirl was built and an axisymmetric two-dimensional CFD model was implemented in the same geometry. The combination refers to the specification of the boundary conditions for the numerical model from measurements of the flow field obtained at the same location. The computational domain is also defined to precisely match the burner geometry. Accurate flow components at the inlets, measured through the experiments, are applied as boundary conditions to the simulated model. Detailed combustion kinetics and soot mechanisms are used to model the dynamics of the reacting flow, including soot formation, oxidation, particle size growth, and size distribution in a realistic geometry and flow field. To model the biogas composition, a mixture of methane and carbon dioxide is selected for simplicity in this initial modeling approach. Oxygen is selected as the oxidizing agent, diluted with CO_2_, as oxycombustion is more common in secondary gas treatment, and CO_2_ is one of the species present in the raw gas with a typical concentration in the range of 10–19% for different processes.^27^

The experimental and numerical methods are described in the Methodology section. The results from experimental datasets were obtained for both cold and reacting flows, with comparison with numerical simulations presented in the Results and Discussion section.

## 2 Methodology

In this work, a swirling jet flame is investigated both numerically and experimentally. This configuration was selected as it is simple enough to allow for numerical modeling at a reasonable computational cost while still providing a mean to control the effect of the fluid mechanics on the chemical kinetics through the swirl number.

### 2.1 Experimental Section

#### 2.1.1 Experimental Setup

**Figure 1:**
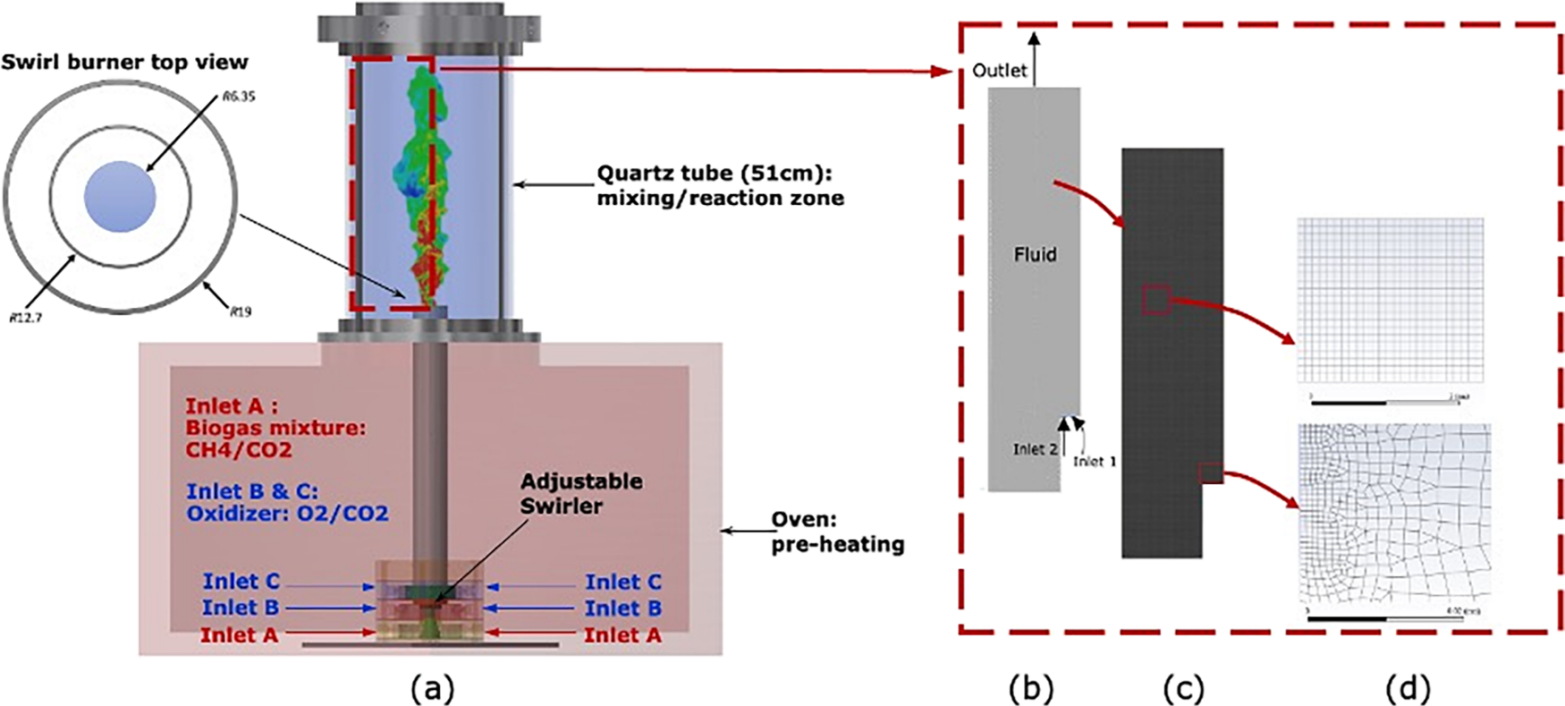
(a) Schematic representation of the modified Cambridge–Sandia swirl burner used in this study, which is enclosed in an oven for reactant preheating; (b) two-dimensional geometry used for numerical studies; (c) entire mesh; (d) close view of the mesh near to and far from the inlet boundaries.

Requirements for an adjustable swirl burner along with the ability to preheat the reactants to high temperature guided the choice of the Cambridge–Sandia swirl burner geometry for this investigation.^28^ Unlike most other swirl burners described in the literature,^29−32^ the Cambridge–Sandia design allows control of the swirl number without moving parts. The whole burner assembly can therefore be placed in an oven as shown in Figure 1a, and the swirl number is changed from the outside using flow controllers, adjusting the gas flow rate sent through the different channels of the burner. The burner consists of two cylindrical annular channels, one where the gas injection is purely axial (inlet A in Figure 1a), surrounded by a swirling channel (inlets B and C) where the gas can be injected either axially or tangentially. The swirl number is changed through the ratio between the axial and tangential flow rates in the swirling channel. Compared to the original Cambridge–Sandia burner geometry, two modifications were made in the present study. First, all tubes were shortened by 100 mm to extend the swirling number range. Second, thin separators were added between the tubes to achieve a consistent alignment and better flow field uniformity.

The combustion takes place in a transparent quartz chamber to enable access for optical diagnostic tools. The burner has an axisymmetric geometry, providing the benefit of allowing 2D velocity measurements to be used to reconstruct the 3D flow fields, thereby significantly reducing experimental complexity. The region of highest interest is found from the burner exit to 100 mm downstream, where gas mixing and heat release occur followed by partial oxidation; soot formation is thought to begin in this area.

To enable a comparison between experiment and numerical simulation, the boundary conditions of the flow field were carefully assessed using Particle Image Velocimetry (PIV) in cold flow conditions. The swirl number of the burner was also characterized from these cold flow measurements. The PIV technique consists of a pulsed laser (Nd:YAG), a double-framing camera, optical lenses, and an aerosol generator to seed the flow (TOPAS ATM 221). Image processing and analysis was performed using the DaVis software from LaVision.

#### 2.1.2 Cold Flow Characterization

The flow swirl improves the mixing of gases, extending the flame stability limits. To characterize the swirl behavior, a combination of 2D velocity fields, acquired at the burner upstream boundary, was used to calculate the swirl number. Equation 1, as specified by Chigier and Beér,^33^ was used for calculating the swirl number, where *R* is the radius of the outer inlet annulus and *w* and *u* are the tangential and axial velocity components.
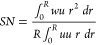
1

The axial velocity component, *u*, is extracted from a 2D velocity field taken in a plane perpendicular to the burner axis. The tangential velocity component, *w*, is extracted from the velocity field taken in a plane parallel to the burner axis. The PIV measurements used to characterize swirl number are acquired at an axial location 5 mm above the burner exit, captured by a lens with high magnification and a narrow field of view (*f* = 105 mm, Nikon), which allows for a fine spatial resolution close to the burner exit.

#### 2.1.3 Reacting Flow Conditions

For the reacting flow cases investigated, methane was used as a fuel, and oxygen was used as an oxidizer. Both the fuel and the oxidizer were diluted with carbon dioxide. The fuel mixture was injected to the inner channel, and the oxidizer mixture was injected through the outer channel, as it requires a larger cross section to achieve the desired velocities. A range of fuel concentrations was investigated, with mixture strengths ranging from 0.46 to 1.15 to cover lean, stoichiometric, and rich conditions. The swirl number was also varied to cover low-, medium-, and high-swirl conditions.

All flow conditions considered here are listed in Table 1. Note that the mixture strength φ defines the stoichiometrically weighted ratio of the fuel mass flow rate to the oxidizer mass flow rate in both annular inlets of the burner, where all cases at φ < 1 and at φ > 1 are fuel-lean and fuel-rich, respectively.

**Table 1:** List of Reacting Flow Cases

case number	*SN*	inner flow rate (L/min)	methane flow rate (L/min)	total outer flow rate (L/min)	oxygen flow rate (L/min)	mixture strength, φ
c1	0.29	5	2	10	8	0.46
c2	0.29	5	3	10	8	0.69
c3	0.29	5	4	10	8	0.92
c4	0.29	5	5	10	8	1.15
c5	0.51	5	2	10	8	0.46
c6	0.51	5	3	10	8	0.69
c7	0.51	5	4	10	8	0.92
c8	0.51	5	5	10	8	1.15
c9	0.82	5	2	10	8	0.46
c10	0.82	5	3	10	8	0.69
c11	0.82	5	4	10	8	0.92
c12	0.82	5	5	10	8	1.15

To assess the effects of the flame chemistry and swirl number on soot formation, soot particle size distributions and concentrations were measured using a Scanning Mobility Particle Analyzer Sizer (SMPS) (TSI Incorporated Model 3980). The particle size range that can be detected with the operating conditions implemented is between 9 and 311 nm. This range covers the vast majority of particles that are present downstream of the reaction zone, but very small incipient soot particles that might form in lean combustion conditions could not be detected experimentally. Although the presence of such small particles might be relevant in some applications, they have a negligible impact on the total mass of particulate matter generated by the flame. When such particles are of interest, the modeling tools evaluated here can be a valuable option to overcome the limitations of experimental tools to detect very small particle sizes.

The SMPS measurements were carried out well downstream of the reaction zone 60 cm from the injection of the burner nozzle, corresponding to approximately two times the flame height. The flow was continuously sampled through a 4 mm inner diameter stainless steel tube with sample flow rate of 0.3 L/min and sheath flow rate of 8 L/min, resulting in conditions in the sampling line where aggregation is expected to be negligible.^23^ However, the sampling approach implemented in the present work does not allow the mapping of soot concentration and size distribution in space or time; the measurement collected provides only an average value of the soot characteristics downstream of the reaction zone.

### 2.2 Numerical Analysis

ANSYS Workbench and ANSYS Meshing were used for preparing the geometry and building the computation grid, respectively. ANSYS Chemkin-Pro was used in preparing, combining, and reducing the chemical reaction mechanisms to be coupled with the CFD in the ANSYS Fluent.

#### 2.2.1 General Flow Model

**Figure 2:**
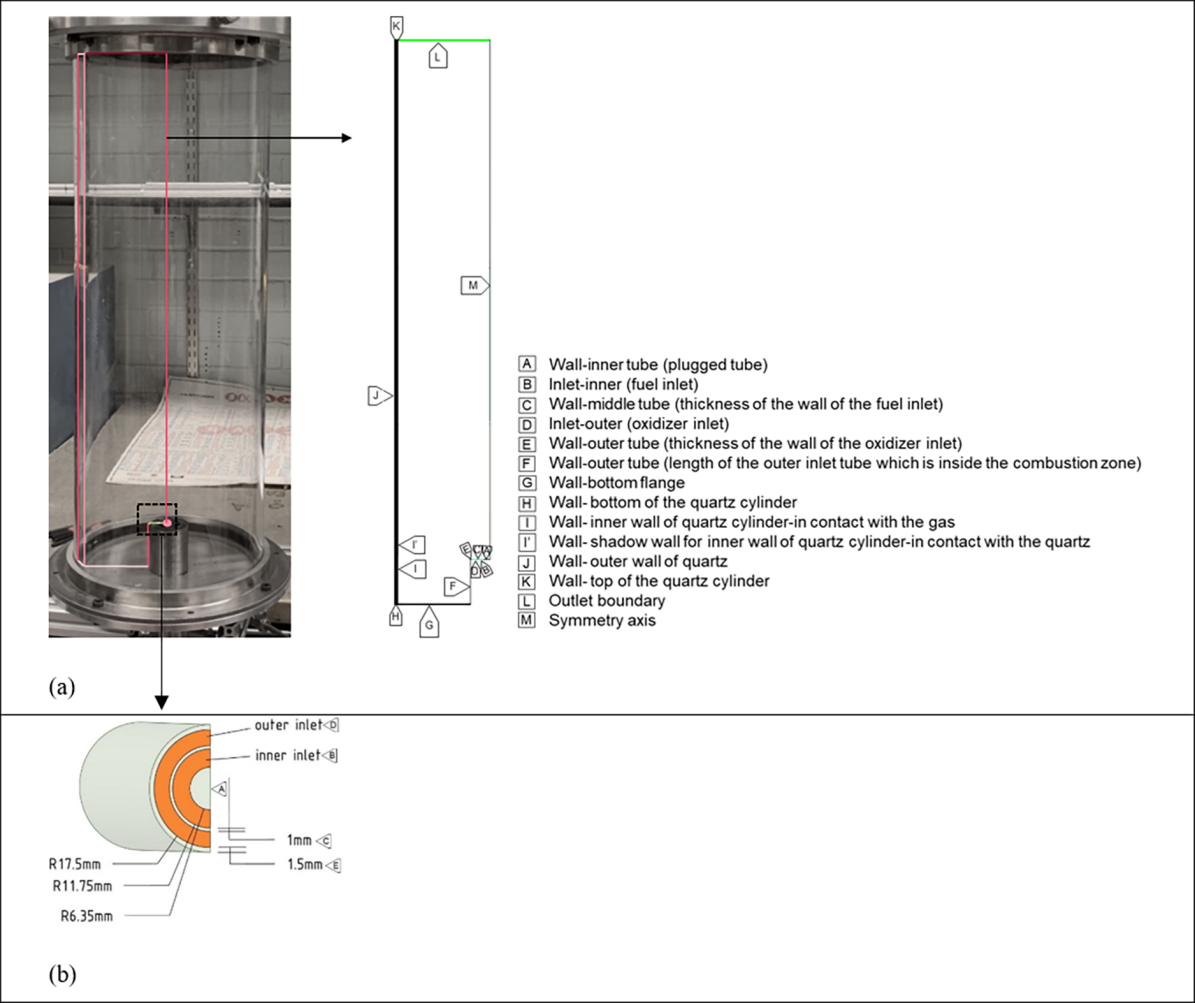
(a) Geometry of all boundaries and (b) dimensions of the inlet boundaries in mm.

A two-dimensional (2D) axisymmetric geometry is considered as shown in Figure 1b–d. The symmetric nature of the flow field inside the reaction zone was verified against the PIV results with no significant azimuthal variations. The dimensions of the inlet boundaries are shown in Figure 2b, with detailed parameters defined in Figure 2a and reported in Table S1.

No curvatures and complex elements exist in the burner geometry, as shown in Figure 1b, and therefore equilateral or equiangular elements, i.e., quad mesh, could be used to create the grid. Three different grids, based on the maximum mesh sizes of 0.4, 0.3, and 0.2 mm, were constructed. To achieve a better resolution around the inlet boundaries, a finer mesh of 0.05 mm was implemented locally. A comparison between the meshes in terms of number of nodes and elements, the mesh quality, and the standard deviation of mesh quality is given in Table 2. To find a suitable compromise between mesh size and computation requirements, the meshes were pre-evaluated and compared with experimental results for the nonreacting cases. Two mesh sizes (0.3 and 0.2 mm) were able to capture the results well, and the finer mesh was used in all following CFD cases. This is important for reacting flow simulation, where details in the spatial distribution of the reaction rate can be captured more accurately with the finer mesh shown in Figure 1c–d.

**Table 2:** Mesh Data for Three Differently Constructed Meshes

max element size (mm)	number of nodes	number of elements	element quality, average	element quality, standard deviation
0.4	222,972	219,476	0.9218	0.23793
0.3	384,147	379,505	0.93938	0.21044
0.2	802,312	799,971	0.9829	0.064251

The experimentally measured flow fields at the inlet boundaries were used as input for the model boundary conditions. The axial (*u*) and radial (*v*) velocity components for the inner inlet boundary followed a parabolic distribution along the radius of the jet. For the outer annular (swirling) inlet boundary, the measured velocity component (*w*) deviated from a purely parabolic and symmetric distribution, with the maximum velocity located toward the outer wall. Functions were fitted to these experimentally measured velocity profiles and used as boundary conditions in the numerical model through user-defined functions (UDFs).

The Transition SST model with standard constants^34^ was applied to model the flow and turbulence. The model is based on the coupling of the κ–ω transport equations (two equations) with two other transport equations, one for the intermittency and one for the transition onset criteria, in terms of the momentum–thickness Reynolds number. Two other methods, RSM (Reynolds stress model) and RNG k-ε (renormalized group k-epsilon),^34^ were compared to the SST model on a cold flow case, and the results showed that the Transition SST has much better agreement with the experimental results. A comparison between the k-ε and the SST model on the reacting flow also supported the choice of the SST model, although the k-ε turbulence model showed a smoother convergence. A comparison of mean velocity contours between three turbulent models and the experimentally measured data is available in Figure S1 in the Supporting Information.

#### 2.2.2 Reactive Model

A reaction mechanism based on GRI-Mech 3.0 [6] was coupled to the flow model to account for the gas phase combustion reactions. Since the oxidizer stream excluded nitrogen, a reduced version of the GRI-3 mechanism (36 species and 219 reactions) was prepared using Chemkin-Pro (see files in the Supporting Information). To obtain soot particle distribution information, the Method of Moments^35^ was used with three moments applied.

To account for radiation effects, a semitransparent model for the walls was implemented. Mixed radiation, conduction, and convection heat transfers were included in the modeling of the outer wall of the burner. Interactions between soot formation, turbulence, and radiation are also included in the CFD model. The effect of soot on radiation is considered by its effect on the absorption coefficient. A summary of all numerical settings and models is given in Table S2.

Mesh independence analysis was conducted first through examining residuals for all equations (momentum, turbulence, species, soot, energy, and radiation) decreased to 1e^–6^. Then, stability of the values of interest was examined at monitor points and along defined lines, as well as along their contours in the whole zone, to ensure reliable results. The monitored values consisted of concentrations of main species and radicals CH_4_/O_2_/CO_2_/H_2_O/OH/C_2_H_2_, temperature, and flow field. Furthermore, the net flux imbalance through the domain boundaries was assessed, which was at an accepted level of 0.5% imbalance.

## 3 Results and Discussion

### 3.1 Cold Flow

**Figure 3:**
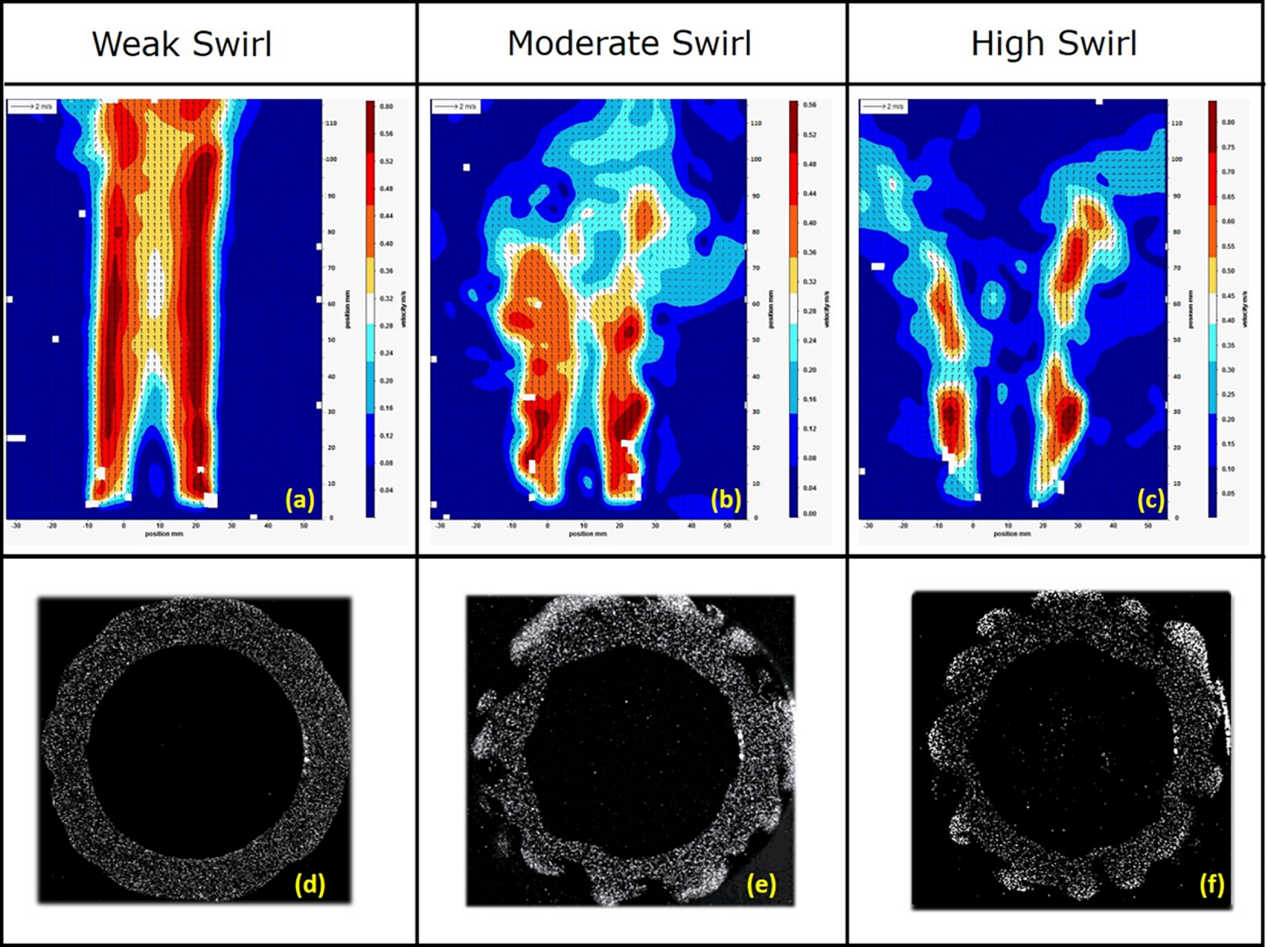
Global flow field behavior with different swirling level ((a, d) weak swirl, *SN* = 0.29; (b, e) moderate swirl, *SN* = 0.51; (c, f) high swirl, *SN* = 0.82). The top row is the front view. The bottom row is the top view.

Figure 3 shows the global flow field obtained from PIV measurements. The dimension of field of view is 80 mm × 110 mm, allowing the observation of large-scale features in the flow field.

As shown in Figure 3a, with a weak swirl (*SN* = 0.29), the flow field remains largely uniform as an annular jet in solid rotation. In Figure 3b, when increasing the swirl intensity, shear layer instabilities appear at the interface between the swirling and axial inlets, and the jet opens at a height of approximately 60 mm. When the swirling magnitude is at its maximum (*SN* = 0.82), as shown from the top view in Figure 3f, a recirculation zone appears approximately 80 mm above the burner exit plane in the middle of the jet.

**Figure 4:**
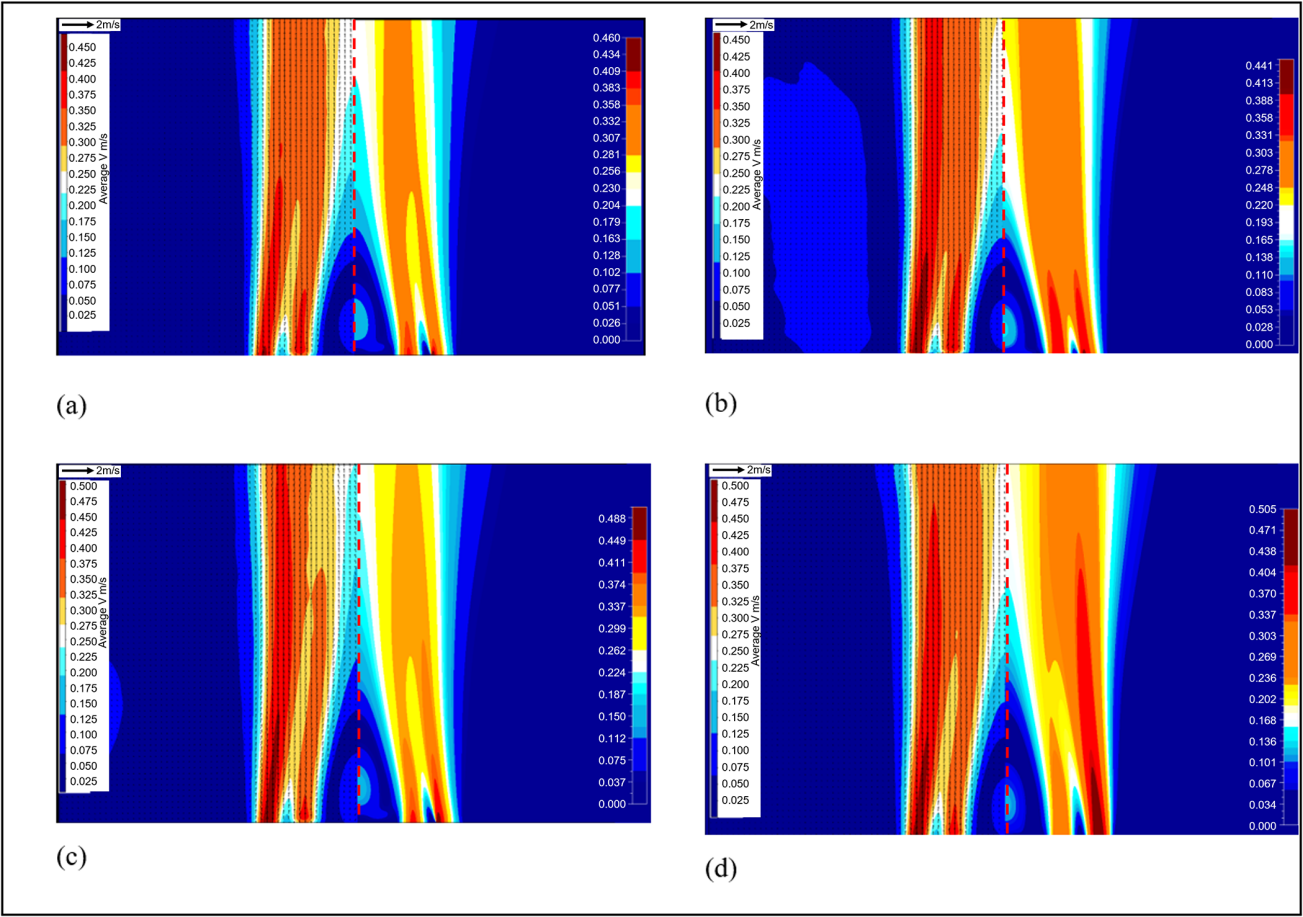
Comparison of 2D longitudinal velocity field between experiments and numerical results for respective cases: (a) case *SN* = 0.13; (b) *SN* = 0.22; (c) *SN* = 0.26; and (d) *SN* = 0.31.

**Figure 5:**
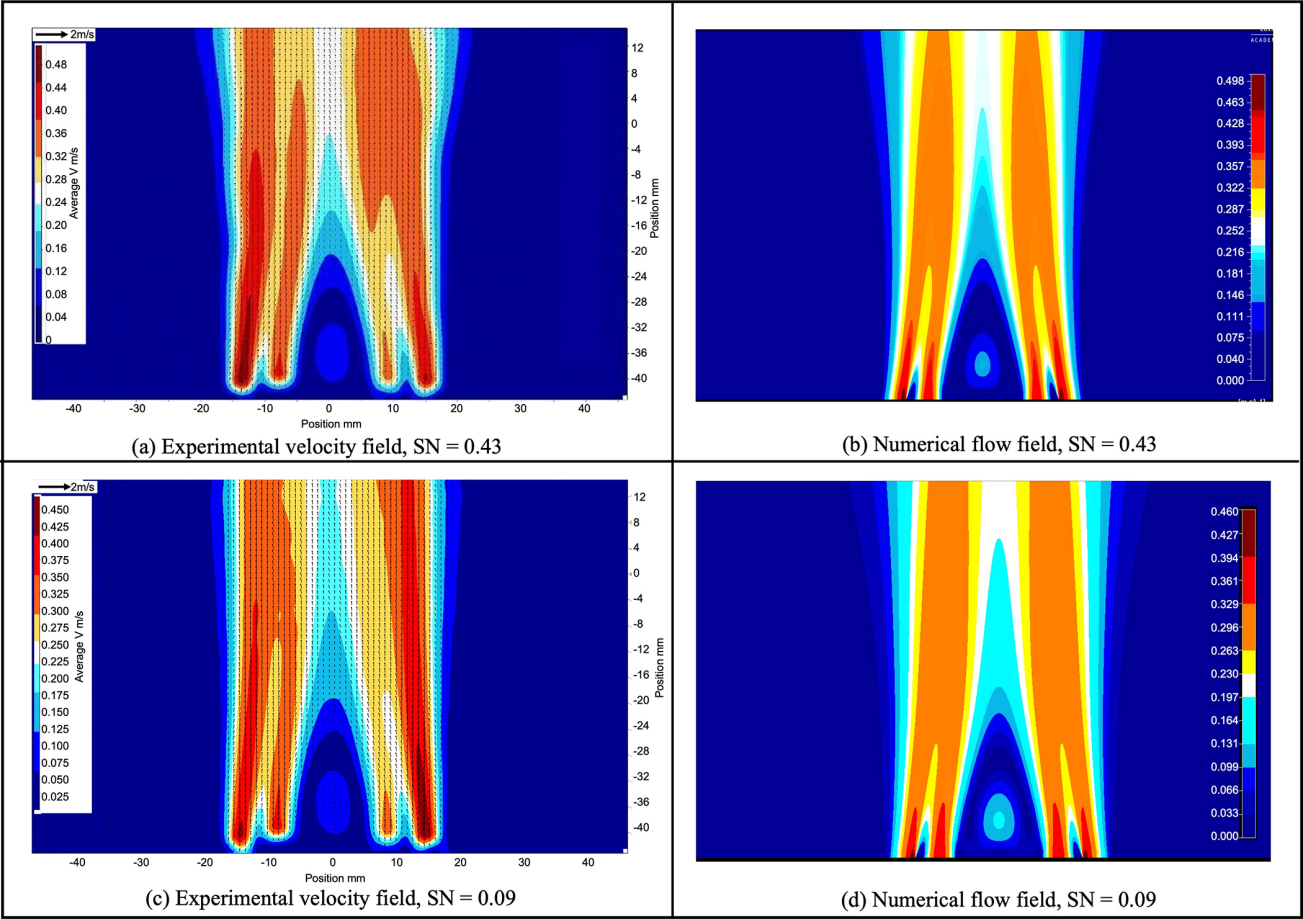
2D velocity fields (m/s) showing slightly transient asymmetry in two of the experimental cases compared to their respective computational results.

The comparison of cold flow fields from PIV measurements and CFD simulations from different cases are reported in Figure 4. In each figure, experimental results (left) and CFD simulations (right) are placed side by side. Overall, they agreed with each other very well; some minor differences are caused by the following possible reasons. First, the color scales are slightly different as the numerical results provide more resolutions for the velocity. Second, in some experimental results, a slight asymmetry is observed between the two sides of the burner as shown in Figure 5. This asymmetry could be caused by slight misalignment of the coaxial tubes comprising the burner inlet. When using experimentally measured velocity profiles as boundary conditions for the numerical model, the average value of the axial component measured on both sides of the jet is used. For the tangential component, the information is extracted from transverse velocity fields, and any asymmetry caused by misalignment is therefore reflected in the boundary. The numerical results are close to an average between the right and left side of the burner. The asymmetry is higher in cases with large flow difference between *u* and *w*, such as cases *SN* = 0.09 and *SN* = 0.43. The characterization cases gathered in cold flow are used as input parameters for reacting flow calculations.

The swirl number in the burner depends exclusively on the total flow rate and the ratio of the axial to the tangential flow rate in the swirling annular inlets, with the other axial channel also contributing to the total flow rate. To investigate the effect of those two parameters, the analysis presented in the following covers three datasets—two datasets with constant system total flow rates of 10 and 15 L/min and one dataset with variable total flow rates.

**Figure 6:**
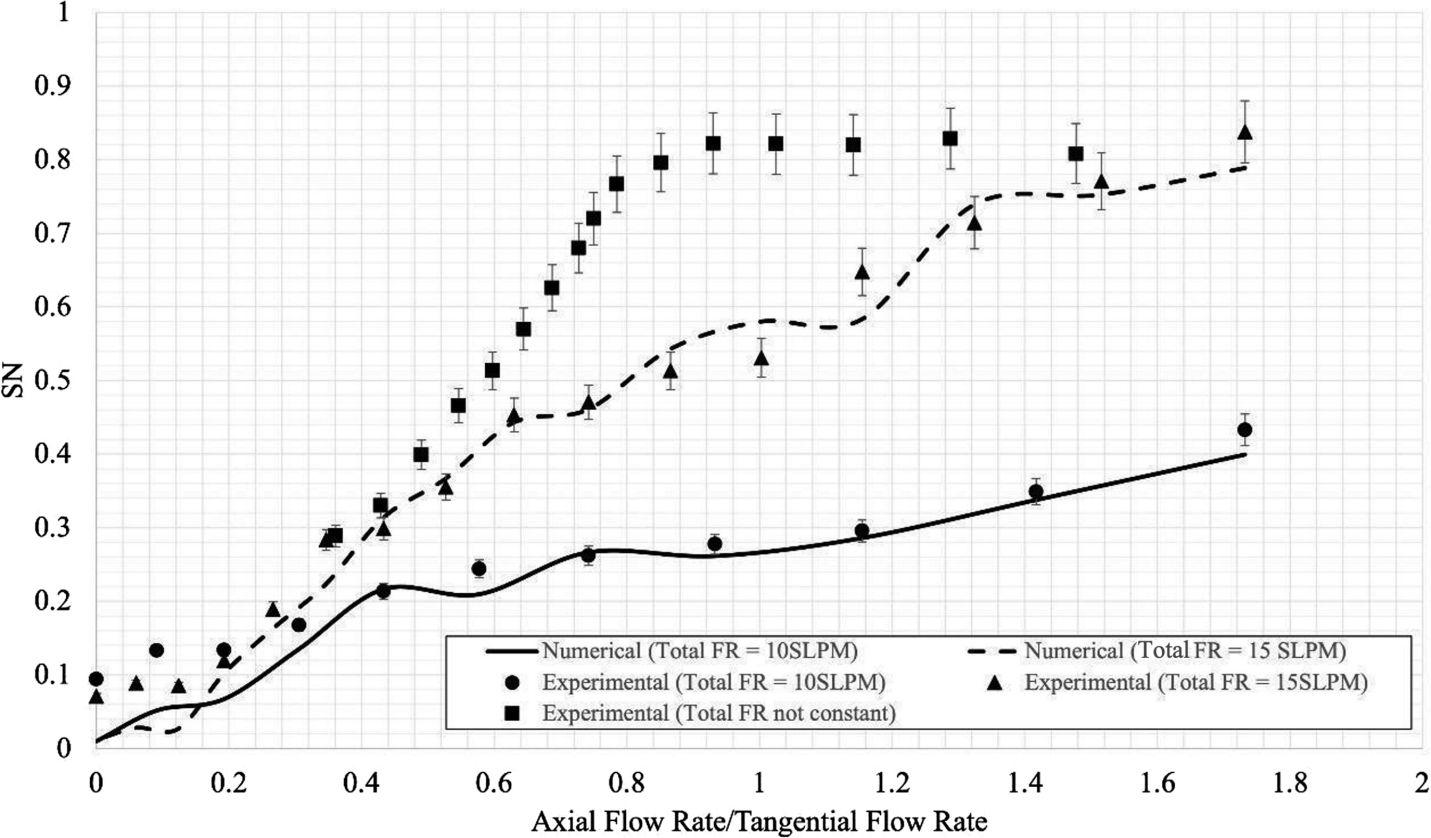
Characterization of swirl number for different total flow rates; experimental results are shown with points and numerical results are shown in lines.

Numerical swirl numbers were calculated as described in Section 2.1.2 using the 2D flow field in a plane 5 mm above the inlets. The results from the experiments and simulations (Figure 6) are in good agreement, demonstrating that the user-defined functions used as boundary conditions in the numerical model adequately represent the inlet flow.

When the total flow rate is kept constant in the swirling channel, the swirl number increases approximately linearly with the ratio of axial and tangential injection flow rates. A higher swirl number can be achieved up to a point with higher total flow rates. Note that the availability of a dataset with a constant flow rate in the swirling channel is important to study the effect of swirl number at constant mixture strength.

When the total flow rate is varied along with the ratio of tangential to axial flow injection, to maximize the swirl number, a plateau is observed with a maximum value of approximately 0.8. Conclusively, the experimental configuration is therefore suitable for covering situations typically referred to as weak (*SN* < 0.3), moderate (0.3 > *SN* < 0.6), and high (*SN* > 0.6) swirl levels.

### 3.2 Reactive Flow

#### 3.2.1 Measurement of Soot Formation

**Figure 7:**
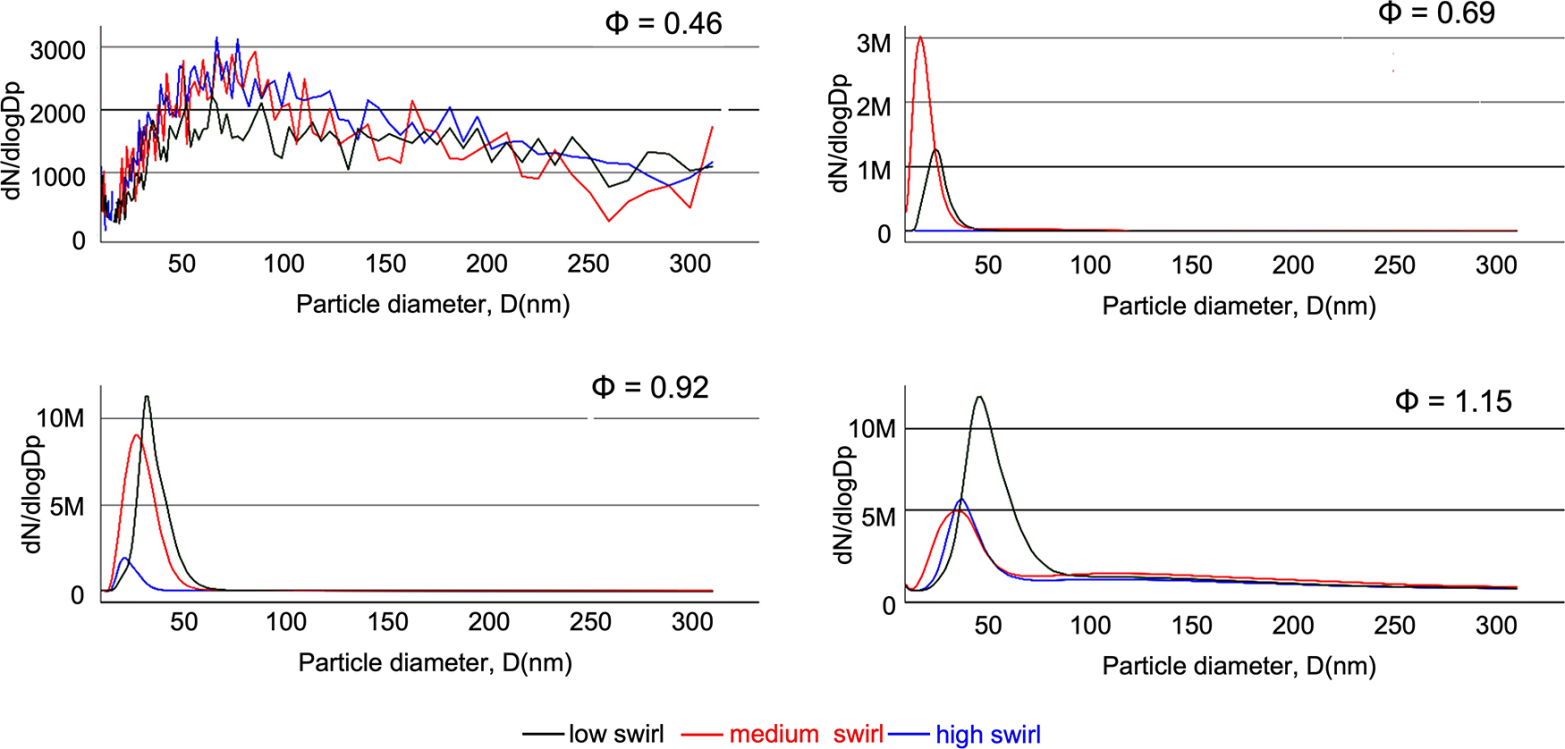
Experimentally measured soot size distribution for different reacting flow cases, where *N* = soot particle number density (particles/m^3^), and the normalized number concentration, d*N*/dlog*Dp*, is calculated by dividing d*N* by the geometric width of the size channel.

Figure 7 shows the results from these SMPS measurements as a function of mixture strength and swirl number. When burning under very fuel-lean conditions (φ = 0.46), soot formation for all swirl number cases is very low, which is less than 0.1% of the values measured under the other fuel conditions. Considering the background aerosol measured in the lab, measured without a flame in the chamber, soot formation under such ultra-lean conditions can be disregarded. The other combustion cases, φ = 0.69, 0.92, and 1.15, exhibit rather narrow size distributions, with particle sizes from 9 nm (the lower detection limit) up to 80 nm. This is close to the size range of 20 to 60 nm (standard deviations of 15–25%) typically observed for primary soot particles,^36^ which are the building blocks for larger aggregate particles formed continuously^37^ and are only visible in our measurements in low numbers for the richest flame condition. Particles with diameters over 311 nm or below 9 nm are therefore not expected in significant amounts under the test conditions investigated.

A significant effect of the swirling flow fields can be observed for all other burning conditions (φ = 0.69, 0.92, and 1.15) in Figure 7. In general, the highest swirl number results in lower soot particle concentration and particle sizes. This is potentially due to a better mixing between the fuel and the oxidizer provided by the swirl, resulting in a more efficient combustion process and better soot oxidation and, thus, a reduced production of smaller soot particles.

For lean flames (φ = 0.69), the swirl is very effective at mitigating soot formation. Under those conditions with high swirl number, the flames are almost soot-free in sharp contrast to the measurement at low or medium swirl number. For close-to-stoichiometric and rich flames (φ = 0.95, φ = 1.15), although the swirl still reduces the amount of soot present, there is no such drastic suppression of soot formation. High and medium swirl numbers, however, still appear to yield similar effects at higher mixture strength, reducing the production of soot particles although only to a certain extent. Considering that acquiring such experimental datasets is very time-consuming, only representative cases were investigated. To cover flame conditions ensuring an adequate validation of the numerical model, a 3 × 4 full factorial experimental design was implemented to assess the effect of swirl intensity and mixture strength.

#### 3.2.2 Numerical Simulation of Soot Formation

**Figure 8:**
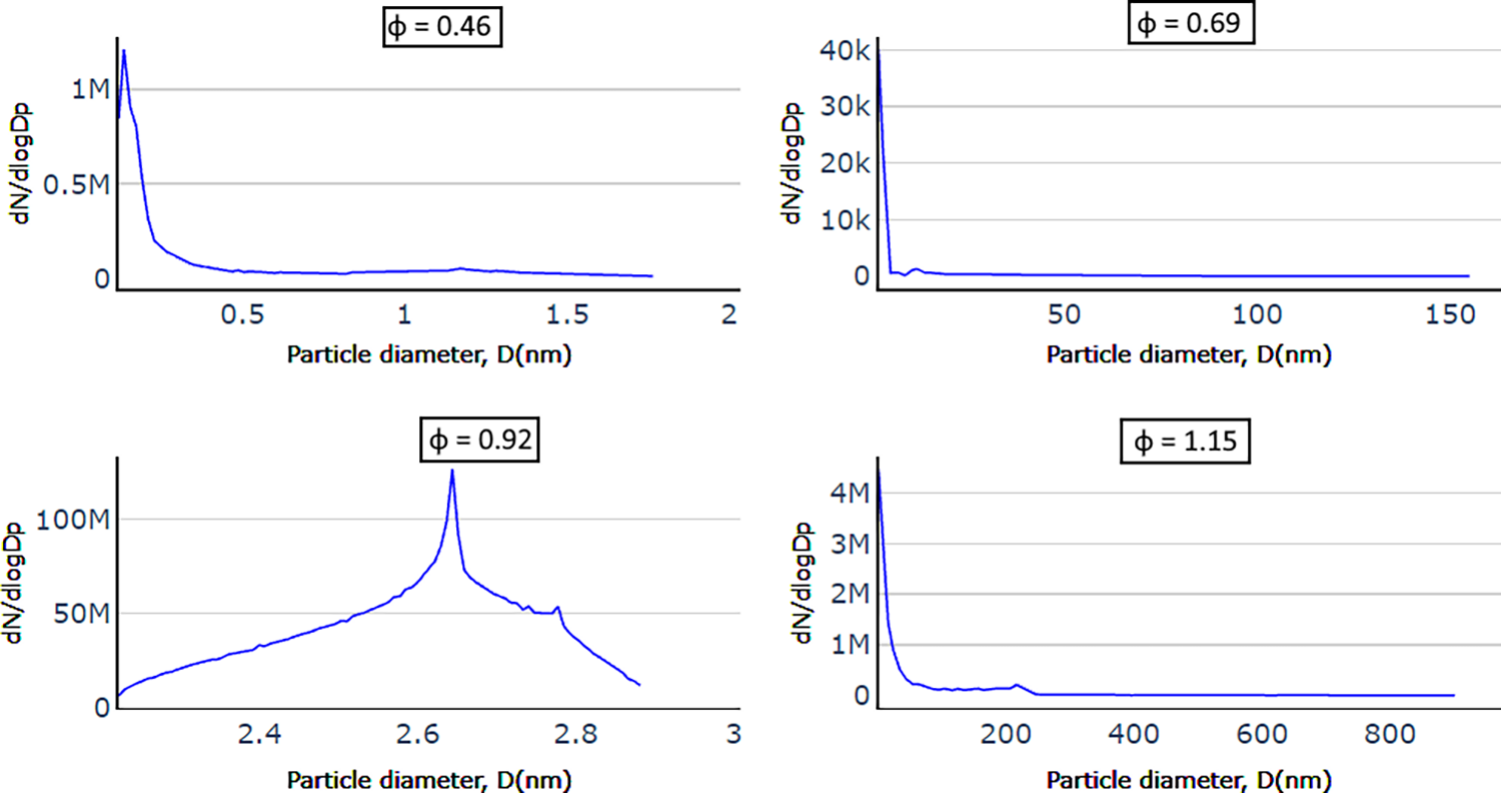
Particle size distribution obtained from the numerical model for high-swirl cases (cases c9–c12); results for different mixture strengths 0.46, 0.69, 0.92, and 1.15.

Figure 8 shows the numerically calculated soot particle size distributions and number density for different mixture strengths for high-swirling intensity cases c9–c12 evaluated at the location where the experimental data was taken. The trend for the growth of the soot particle size as a function of the fuel strength is similar to the trend observed in the SMPS measurements, with progressively richer flows yielding larger particles, ultra-lean (φ = 0.46) < stoichiometric (φ = 0.92) < fuel-lean (φ = 0.69) < fuel-rich (φ = 1.15). This is an expected result since all fuel-lean cases with φ < 1 could achieve complete combustion. The numerical results capture well that such lean flames have a very low amount of soot formation and are supported by the experimental results, where insignificant amounts of soot were measured for particle sizes above 9 nm. For the fuel-rich case (φ = 1.15), the particle sizes from the numerical results show growth up to 900 nm, which are much larger particle sizes compared to the limit value of 311 nm that could be measured by the SMPS in the configuration used in the experiments. However, the amount of such large particles is insignificant compared to the total amount. Thus, this is not the most interesting region of this study, since most of the particles are within the region below 200 nm.

**Figure 9:**
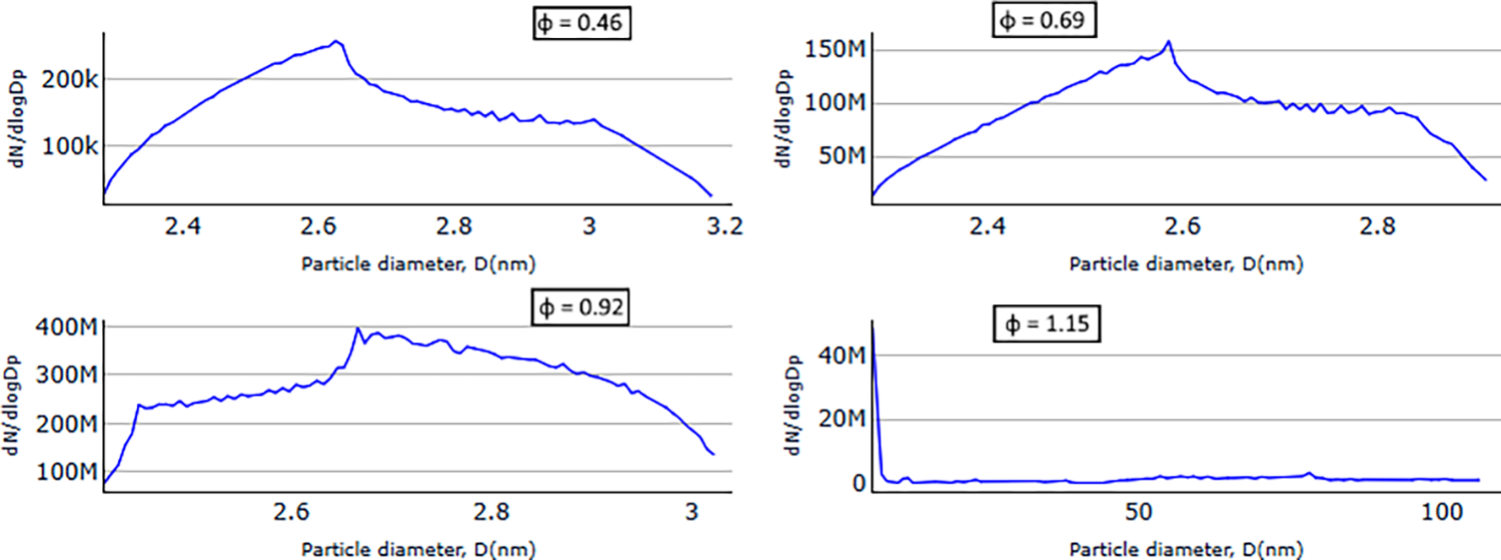
Particle size distribution obtained from the numerical model for medium-swirl cases (cases c5–c8); results for different mixture strengths 0.46, 0.69, 0.92, and 1.15.

**Figure 10:**
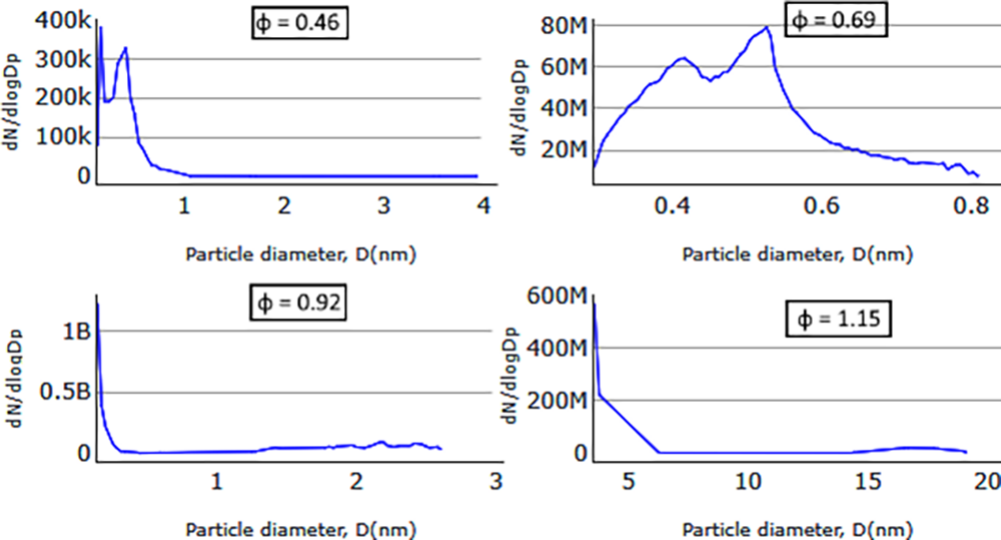
Particle size distribution obtained from the numerical model for low-swirl cases (cases c1–c4); results for different mixture strengths 0.46, 0.69, 0.92, and 1.15.

Comparing soot particle sizes obtained from the numerical simulations and the experimental measurements for the fuel-lean cases, φ < 1, reveals that the simulations predict particles much smaller than those observed in the experiments. This can be due to either an underestimation of the soot growth rate or an overestimation of the soot oxidation rate in the numerical model. The latter case appears to influence the results more substantially. When looking at soot growth over the entire combustion chamber, the numerical results reveal the existence of very large soot particles in the range of 150 to 230 nm formed for cases c3, c7, and c11. These large particles are subsequently oxidized and therefore not detected 60 cm above the nozzle, where the comparison with the experiments is carried out. As a result, the average particle sizes at the outlet sampling point for fuel-lean cases remain below 5 nm (Figures 8–10). This discrepancy is most likely caused by an overestimation in the model of the radical concentration (O and OH) in the postflame region of the combustion chamber. These radicals are known to rapidly oxidize soot.^24,38−41^ The cause of this overestimation is, however, not known but could originate in the boundary conditions used for radiation or in the reduced chemical kinetics used. This could be better understood by the validation of concentrations of radical species in this zone against experiments. Unfortunately, the present experimental setup does not have this possibility. For fuel-rich cases such as c12 (shown in Figure 8), there is no possibility for the model to overestimate the concentration of radical oxidants (O and OH) in absence of excess oxygen; consequently, the numerical results tend to exhibit similar trends to those observed in the experiment.

Soot particle size distributions predicted numerically for medium-swirling flow fields for different mixture strengths are shown in Figure 9, revealing a mode heavily skewed toward much smaller particles compared to what was observed experimentally. Keeping in mind that the SMPS does not allow measurements of particles smaller than 10 nm, the main peak shown in the simulation results could therefore not be captured experimentally. For the fuel-rich case (Figure 9), the soot mean diameter () among the sampled numerical particles is 44 nm. This is slightly smaller compared to 57 nm from the experimental results. However, the maximum diameter for soot particles is 178 nm, which is significantly smaller than the experimentally measured size of 311 nm. One potential factor that affects the numerical soot sizes is the average soot particle density of 1800 kg/m^3^ used for all numerical cases, which is the most often used value in the literature.^26^ This density is more relevant to fuel-rich cases, and therefore, particle sizes deviate more from experimental results in fuel-lean cases.

The soot particle size distributions obtained from the numerical model for low-swirl flow fields for different mixture strengths are shown in Figure 10. For all φ, the soot particle sizes predicted by simulations are much smaller than those observed in experiments. This is even seen for the fuel-rich case, φ = 1.15, in contrast with the moderate- and high-swirl cases described above and shown in Figures 8 and 9.

**Figure 11:**
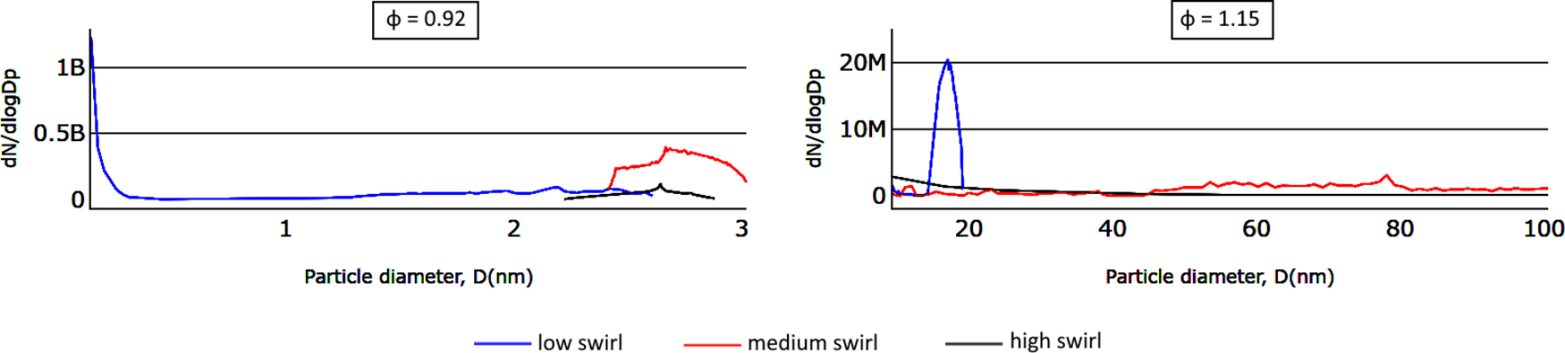
Particle size distribution obtained from the numerical model as a function of swirl number for mixture strengths 0.92 and 1.15.

The effect of swirl on numerically predicted soot particle size is shown in Figure 11 for φ = 0.92 and 1.15. Comparing soot number density between medium- (red) and high-swirl (black) flows, it is seen that increasing the swirl helps in soot number reduction for both cases.

The comparison between numerical and experimental results in terms of the soot particle size distribution demonstrates that the numerical model captures well the effect of the flow field. Moreover, these results show that the agreement is better for high-swirl number cases. However, the soot model itself has a poor ability to accurately predict the soot size distribution and number density measured experimentally. The coupling of several numerical submodels in this work to cover combustion kinetics, soot formation, and population development, radiation, and swirling effects showed that even when using very accurate experimental data for the boundary conditions, the simulation tool cannot give a quantitative prediction of soot particle size distributions. Although the commercial codes can be used to assist in the design of partial oxidation reactors, users should be aware of their limitations in the prediction of soot size distribution.

## 4 Conclusions

In the present study, experimental and numerical tools are used to investigate soot formation in a swirling burner. A burner allowing control over the swirl intensity was manufactured and operated to cover weak-, moderate-, and high-swirl conditions for a methane/oxygen flame with variable stoichiometry. The soot particle size distribution was obtained from a scanning mobility particle sizer (SMPS), with results showing that increased swirl has a significant effect in reducing soot concentration and sizes under all burning conditions. For lean flames, swirling is very effective at mitigating soot formation. However, for rich flames, swirl helps reduce the production of soot particles but only to a certain extent.

The numerical modeling of soot particle sizes and concentrations is attempted using a commercial CFD code (ANSYS Fluent), including the effect of turbulence, detailed combustion kinetics, radiation, soot formation, and oxidation. For a few flame conditions, the simulation yielded trends similar to those observed in the experiments, but in general, the numerically simulated soot particle sizes are much smaller than those measured. Possible reasons for these differences include limitations in soot-related submodels and the fact that the available SMPS instrument could not detect particles below 10 nm, a size range for which the numerical model predicted very high concentrations.

The numerical modeling of soot particle sizes has always been a challenging problem. The agreement between the results of the numerical simulation and the experimental measurements could be slightly improved by tweaking the parameters of the soot model, for instance, by using different average soot particle densities for fuel-rich and fuel-lean cases. However, as our results demonstrate, quantitative numerical prediction of soot concentrations and size distributions in complex turbulent flows would require the development and implementation of significantly more advanced models for soot particle nucleation, growth, aggregation, and oxidation.

## Supporting Information Available

(Figure S1) Comparison of mean velocity contour results between (a) experimental and three turbulent model alternatives: (b) transition SST model; (c) RNG k-ε; and (d) RSM; (Table S1) detailed settings of all boundaries for the numerical model; (Table S2) summary of reactive model settings for the numerical model (PDF)
Reduced GRI-3 kinetic mechanism files (36 species and 219 reactions) together with their relevant thermo- and transport files (ZIP)

## ABBREVIATIONS

PIV: particle image velocimetry
CFD: computational fluid dynamics
POX: partial oxidation
SMPS: scanning mobility particle sizer
SN: swirl number
UDF: user-defined function
