# Supplementary material for: Experimental and Numerical Investigation of Flow Field and Soot Particle Size Distribution of Methane-Containing Gas Mixtures in a Swirling Burner
Source: ACS Omega. 2021 Dec 22;7(1):469–79. doi: 10.1021/acsomega.1c04895 (PMC8757356; doi:10.1021/acsomega.1c04895)
Supplement: Supplementary file 1 — ao1c04895_si_001.pdf [file ao1c04895_si_001.pdf]

Supporting Information for

Experimental and numerical investigation of flow field and soot particle size distribution of methane-containing gas mixtures in a swirling burner

*Zari Musavi‡Ψ, Yao Zhang‡Λ, Etienne RobertΛ and Klas Engvall*Ψ*

Ψ KTH Royal Institute of Technology, Dept. of Chemical Engineering, Stockholm, Sweden

ΛPolytechnique Montréal, Dept. of Mechanical Engineering, Montréal, Canada

Supportive information includes the following contents:

Figure S1. Comparison of mean velocity contour results between a) experimental; and three turbulent model alternatives: b) transition SST model; c) RNG k-epsilon; and d) RSM.

Table S1. Detailed settings of all boundaries for the numerical model

Table S2. Summary of reactive model settings for the numerical model

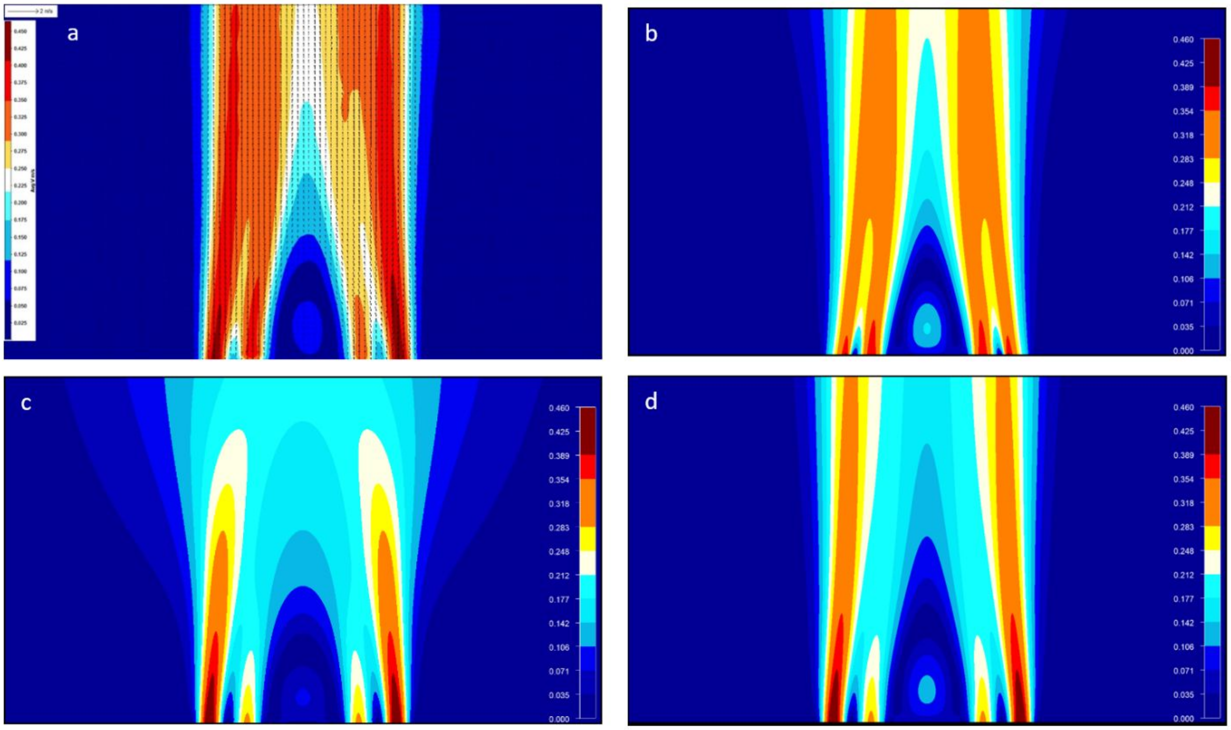

Figure S1. Comparison of mean velocity contour results between a) experimental; and three turbulent model alternatives: b) transition SST model; c) RNG k-epsilon; and d) RSM.

Table S1. Detailed settings of all boundaries for the numerical model

NAME	A	B	C	D	E	F	G	H	I	I'	J	K	L	M
Boundary type	wall	veloc ity- inlet	wall	veloc ity- inlet	wall	wall	wall	wall	wall	wall	wall	wall	outfl ow	axis
Wall Thickness, mm							24.5	24.5						
Material Name	steel		steel		steel	steel	steel	steel	quartz	quartz	quartz	quartz		
Wall Roughness Height (m)	0		0		0	0	0		0					
Wall Roughness Constant	0.5		0.5		0.5	0.5	0.5		0.5					
Thermal BC Type	zero heat [illegible]	Temp. 293K	zero heat [illegible]	Temp. 323K	zero heat flux	zero heat flux	convection	convection	radiation	radiation	mixed: radiation,	convection		
Convective Heat Transfer Coefficient, W/m-K							4	4			8	8		
Free Stream Temperature							293	293			293	293		
Radiation BC Type	opaque		opaque		opaque	opaque	opaque	opaque	semi- [illegible]	semi- [illegible]	semi- transpare	opaque		
Internal emissivity for each band (3	(0.4 0.4 0.4)	0	(0.4 0.4 0.4)	0	(0.4 0.4 0.4)	(0.4 0.4 0.4)	(0.4 0.4 0.4)	(0 0 0)	(0 0 0)	(0 0 0)	(0 0 0)	(0 0 0)	0	

bands are defined)-														
Diffuse fraction for each band	(1 1 1)		(1 1 1)		(1 1 1)	(1 1 1)	(1 1 1)	(1 1 1)	(1 1 1)	(1 1 1)	(1 1 1)	(1 1 1)		
External Emissivity											0.01			
External Radiation Temperature											293			

Table S2. Summary of reactive model settings for the numerical model

Model	Settings
Space	Axisymmetric swirl
Time	Steady
Viscous	Transition SST model
Radiation	Discrete ordinates, 3 non-gray bands
Species	Reacting (36 species- based on GRI-3)(h2 h o o2 oh h2o ho2 h2o2 c ch ch2 ch2<s> ch3 ch4 co ar hco ch2o ch2oh ch3o ch3oh c2h c2h2 c2h3 c2h4 c2h5 c2h6 hcco ch2co hccoh n2 c3h7 c3h8 ch2cho ch3cho co2)
Soot	Nucleation based on acetylene, surface growth based on HACA mechanism, oxidation by oxygen and hydroxyl radical

Discretization Scheme for different variables	Scheme
Pressure	PRESTO!
Momentum	Second Order Upwind
Swirl Velocity	Second Order Upwind
Turbulent Kinetic Energy	Second Order Upwind
Specific Dissipation Rate	Second Order Upwind
Intermittency	Second Order Upwind
Momentum Thickness Re	Second Order Upwind
Species	Second Order Upwind
Soot Method of Moments	Second Order Upwind
Energy	Second Order Upwind
